# Automated Calibration System for Digital Multimeters Not Equipped with a Communication Interface

**DOI:** 10.3390/s20133650

**Published:** 2020-06-29

**Authors:** Grzegorz Grzeczka, Maciej Klebba

**Affiliations:** Faculty of Mechanical and Electrical Engineering, Polish Naval Academy, 81-127 Gdynia, Poland; g.grzeczka@amw.gdynia.pl

**Keywords:** automation, calibration, dot recognition

## Abstract

This article is focused on the calibration of digital multimeters, in which the concept and practical solutions for stations with software for automatic calibration are presented. This paper also presents the general structure of the measuring system, the application scheme, and the technical implementation of measuring stations, together with the software.

## 1. Introduction

**Figure 1:**
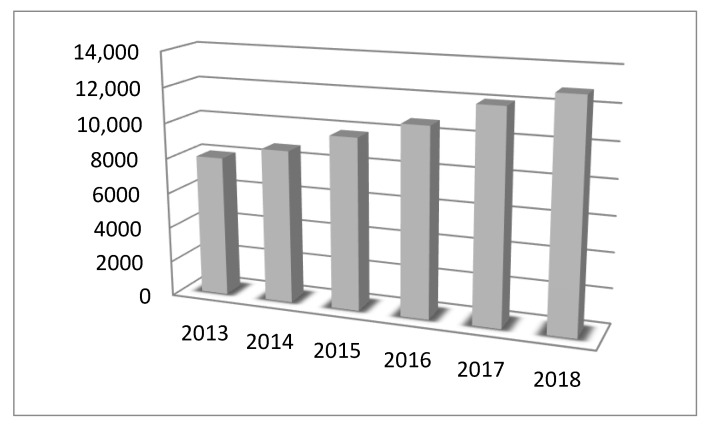
List of the calibration numbers performed in the First Military Metrology Center in 2013–2018.

In light of the dynamic technological development that is taking place at present, the proper operation of technical devices is an extensive and complicated task. One of the key elements that ensures the proper operation of devices is the measuring and monitoring of their most important parameters. To be able to properly carry out diagnostics and the operation of devices, it is necessary to rely on reliable measuring instruments. This reliability can be confirmed by periodically checking the technical condition of the measuring instruments in the process of calibration or verification. The form of metrological control depends on the type of instrument and its intended use [1]. In the case of instruments intended for military purposes, the applicable regulations require that each measuring instrument is subject to periodic metrological control [2]. The number of measurements carried out increases in line with technological development. At the time of great geographical discoveries, sailing ships that were able to reach virtually any place on Earth required for their functioning a compass, a sextant, and possibly several other simple measuring instruments. Currently, even small vessels are equipped with several hundreds of more specialized measuring instruments. This causes a significant increase in the demand for metrological services [3]. Figure 1 shows the increase in the number of calibrated instruments using the First Military Metrology Center as an example. The information presented in Figure 1 was created on the basis of data from the military metrology information system LOGIS_NET and collected by the author during his work at the First Military Metrology Center in 2013–2018.

As can be seen in the graph, the number of measuring instruments subject to metrological services is constantly increasing. This tendency will probably continue or increase in the coming years. To meet these challenges, metrology takes actions to improve work efficiency. One of the methods to increase work efficiency is to introduce measurement automation [4]. In the modern world, as a result of automation, measuring equipment is becoming increasingly more complicated and more expensive, but much simpler to use [5]. This results in dynamic development and the introduction of an increasing number of new measuring methods using systems or devices, as well as a significant degree of automation.

Automation of the calibration process applies to both civilian [6] and military institutions [7]. Instruments for measuring virtually all common physical quantities, such as mass [8], resistance [9], as well as specialized instruments such as AC/DC transfer standards [10] and power meters [11], are subjected to the automation process. From the point of view of this article, the most interesting group of devices are digital multimeters. Digital multimeters are one of the largest groups of measuring instruments used for military purposes. For these instruments, it is necessary to determine the measurements for many ranges of different physical quantities, which is a time-consuming and labor-intensive activity [12].

To simplify and shorten the service time, various methods of automating this process are introduced. For example, in the First Military Metrology Center, MET/CAL [13] and Calpro 101 [14] software is used, which allows semi-automatic and fully automatic calibration of some types of digital multimeters. Multimeter calibration software is also created either completely proprietary [15] or based on existing solutions such as LabView or LabWindows/CVI [16].

As can be seen from the examples cited, automating the calibration process is not a new concept, in contrast, it is widely used and brings measurable effects. At the same time it must be remembered that a prerequisite for performing such calibration is that the checked multimeter has a communication interface that allows reading the measurement results [17,18].

To eliminate this limitation, many concepts and solutions based on computer vision have been created. The concept of using image recognition algorithms to automate the calibration process is also not new. In the global literature, there are articles that describe the process of automatic calibration of both digital and analog instruments [19], and different approaches and algorithms are presented. The basis of most operations is the division of the image into individual segments corresponding to individual digits, although there are solutions that eliminate this inconvenience [20]. Although the articles present algorithms and software solutions, most authors do not state how effective they are [21]. These works, where the topic of effectiveness is considered, provide information about the lack of 100% effectiveness [22] or about effectiveness at the level of 99.33% [23] or 99.75% [24]. Although these are relatively high efficiencies, in the solution presented in this article, thanks to focusing only on seven-segment displays, we managed to achieve 100% result recognition efficiency. Due to the fact that a very large group of the digital multimeters used in the army do not have communication interfaces and so far have not been considered as automation objects, the concept of creating a communication interface was developed. Such an interface is able to continuously read the measurement results from the display and to transfer these data to a computer. Furthermore, in this article, the principle of operation and a practical prototype solution are presented. An interface based on computer vision is the basic element of the entire automated calibration system, and the system is adapted to the specifics of the work of military metrology laboratories—including the methods and tools used in the army. The system also meets the requirements of management systems, being compliant with the ISO 17025 standard used in military metrology centers in Poland.

## 2. Materials and Methods

**Figure 2:**
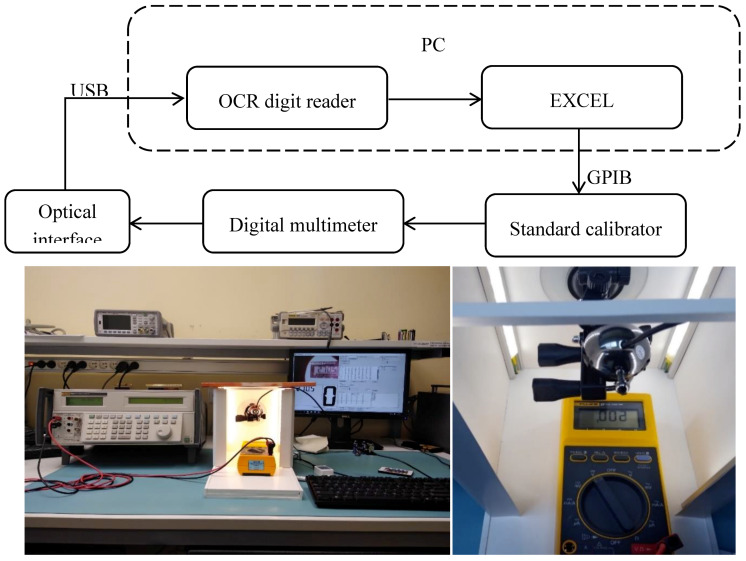
The structure and practical prototype solution of the automatic calibration station.

The calibration process that takes place in the system is shown in Figure 2. The calibrated instrument is connected directly to the standard calibrator. The template is controlled via the General Purpose Interface Bus (GPIB) directly from the form in Excel. The connection of the calibrator to a multimeter is carried out by means of two or four wires connected to the appropriate terminals, depending on the measured physical quantity. The reading of the measurement results from the display is made by means of a camera connected to the computer via a Universal Serial Bus (USB) connection. A camera with a resolution of 1280 × 720 pixels was used in the preliminary tests.

The FLUKE 5500 [25], FLUKE 5502 [26], and FLUKE 5520 [27] multifunction calibrators were used interchangeably. These standards have built-in RS-232 (RS-recommended standard) and GPIB communication interfaces. The RS-232 interface uses serial data transmission, and the transmission speed depends on the length of the connection cable—the typical baud rate is 20 kbps. The disadvantage of this solution is its lack of sufficient protection against interference. GPIB interface uses parallel data transmission [28], which are sent as multi-bit words on the bus lines to which all functional units of the system are connected in any place. The transmission speed is in the range of 1.5–8 MB/s. Due to its undoubtedly better parameters and its greater resistance to interference, the GPIB interface that cooperates with a compatible measuring card installed in a Personal Computer (PC) was used for the tests. The connection of the tested multimeter with the standard was carried out by means of two or four test leads, depending on the measured physical quantity. This connection and changing of the measuring range of the tested instrument were the only activities performed by the technician during the entire metrological service process.

To read the value of the measurement results from the display of the calibrated multimeter, a measuring station with software was created. The OCR Digit Reader (OCR–optical character recognition) is a program that was written by the author to enable quick recognition of the results from seven-segment displays. The program was written in C # [29,30,31,32], which ensures full compatibility with the Windows operating system, and is an operating system commonly used in both calibration laboratories and military metrology centers.

While creating the program, the OpenCV library was generally available [33]. Due to the C # language used, Emgu CV was also used, which is a cross-platform .NET wrapper to the OpenCV image processing library that allows OpenCV functions to be called from .NET-compatible languages. This wrapper can be compiled by Visual Studio [34].

Based on the conducted experiments, it was found that the lighting of the object has the greatest impact on the reliability of the reading. Any change in the intensity and direction from which the light falls on the liquid-crystal display (LCD) may result in the need to change the filter configuration of the OCR Digit Reader program. To minimize the impact of changes and the unevenness of the lighting, a measuring station consisting of a camera, an artificial light source, and housing eliminating the access of external light was built. As a light source, a set of LEDs with white-colored lights (6000 K) and a luminous flux of approximately 1000 lm was used. The stand, measuring 50 × 25 × 22 cm and made of foamed poly vinyl chloride (PVC), was equipped with handles that allowed to place the subsequent calibrated measuring instruments in exactly the same places in relation to the camera capturing the image. The prototype position is shown in Figure 2.

The OCR Digit Reader program is based on the assumption that it is only used to recognize and uniquely identify the image from seven-segment displays. The image is captured using a camera connected to a computer via a USB port. The device must have the ability to send video in real time. A Logitech 1080p camera was used for the research, and various resolutions were tested. At lower settings, due to the smaller number of pixels subjected to analysis, image recognition is faster, but the 1080p resolution reduces the likelihood of ambiguous readings, and the time obtained on an ordinary PC computer at the level of a single millisecond is sufficient for the calibration tasks of measuring instruments.

**Figure 3:**
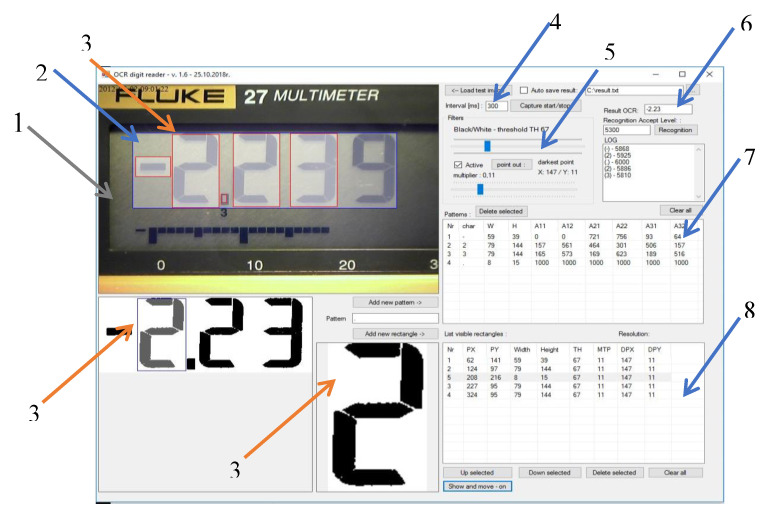
OCR Digit Reader main panel view. (1) The image window captured from the camera; (2) the work area; (3) the frames determining the position of individual characters; (4) the time interval between subsequent readings; (5) the multiplier and black/white filters; (6) the recognition results; (7) the matrix of patterns; and (8) the location, filter sizes, and setting s for individual frames.

Figure 3 shows the program’s communication interface with the main elements marked as (1) the image window captured from the camera; (2) the work area; (3) the frames determining the position of individual characters; (4) the time interval between subsequent readings; (5) the multiplier and black/white filters; (6) the recognition results; (7) the matrix of patterns; and (8) the location, filter sizes, and settings for individual frames.

**Figure 4:**
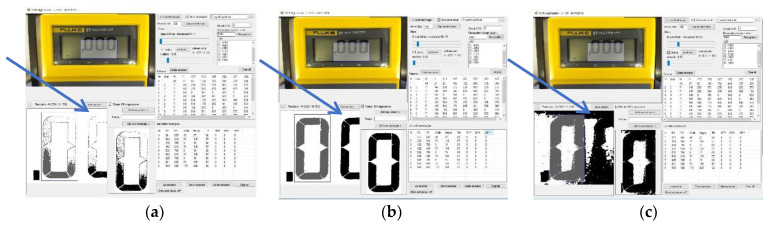
OCR Digit Reader—the effect of using the multiplier and black/white filters. (**a**) too small values (**b**) proper correction (**c**) too large values.

The first stage of the image processing is its conversion into grayscale. Then, using two filters, namely, multiplier and black/white, their parameters are selected so as to obtain a black and white image with the least amount of interference. Noise is considered to be black pixels outside of the field in which the active display segment is located. The effect of the filters is shown in Figure 4.

Figure 4a,c shows a screenshot of the OCR Digit Reader work screen in which the filter settings of both the multiplier and black/white filters are incorrectly selected, respectively. The image in Figure 4c, due to the large number of distortions marked by the arrow, is ambiguous for interpretation. Too many black pixels are located outside of the active area of the individual display segments, which cause ambiguity in specifying the pattern of the individual characters. As a consequence, this setting causes a significant number of misinterpretations and the total inability to interpret the displayed digit or character. Parameter values that are too small cause the absence of black pixels in a large area of the active display segment (Figure 4a). Due to the adopted method of image recognition, this situation causes the individual characters to be characterized by a small percentage of black pixels, despite the active different segments of the display. This situation, despite the lack of interference in the form of additional pixels, makes it difficult or even impossible to unequivocally interpret the displayed numbers. Figure 4b shows a screenshot of the screen, which shows the image after proper correction with the help of filters. To obtain the highest possible efficiency and reliability of the program, during the initial settings, it is necessary to select filter values for the external lighting and the brightness of the display itself, so that the obtained image is free of interference. At the same time, it is important to remember to achieve the highest possible image fill in the active segments of the display.

The next step, when configuring the program to work with a specific type of measuring instrument, is to define the work area, which is a fragment of the whole image captured from the camera and may contain frames that border specific characters. While the program is running, calculations are made only within the work area, where subsequent frames are marked to specify the position of the individual numbers and characters, such as minus signs, periods, or commas. For all possible characters, their patterns are determined and then stored in the matrix of patterns. For each type of multimeter, the program must first be configured and then the data must be saved in the configuration file. Configuration files allow to calibrate subsequent instruments of a single operation type without the need to configure the software each time. The data saved in the configuration files are the following:

- The coordinates of the reading fields;
- The multiplier and black/white filter settings for individual fields and patterns;
- The matrix of patterns;
- The default location to save the results file;
- The default interval time between successive readings; and
- The default acceptable level for a criterion of a reliable result.

The measuring stand included in the whole system, thanks to the possibility of setting a constant value of illuminance, allowed to use configuration files for subsequent calibrations without having to edit them.

**Figure 5:**
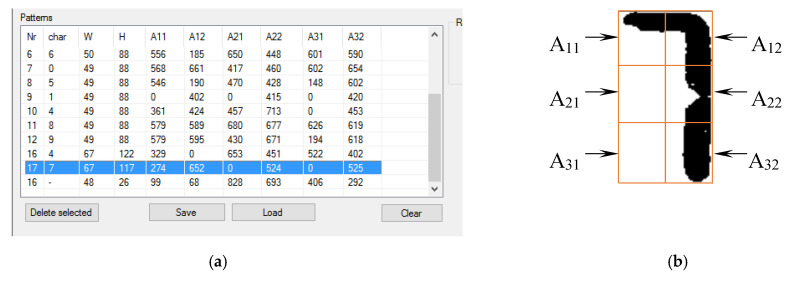
A method for recognizing numbers based on the percentage of black pixels. (**a**) Patterns matrix and (**b**) division into fields.

Character recognition is based on the principle of the percentage count of black pixels in a given fragment of an image. Each readout field is virtually divided into six parts, and the percentage of black pixels is counted in each. In order to simplify further calculations and to work on integer numbers, the calculation result is multiplied by 1000. The result of this operation using the number 7 as an example is shown in Figure 5.

The algorithm for recognizing a single character using the number 7 as an example is presented below. The percentage of black pixels from individual fields after multiplication by 1000 is as follows.

A11 = 274

A12 = 652

A21 = 0

A22 = 524

A31 = 0

A32 = 525

The result matrix obtained in this way is compared successively to all of the patterns found in the matrix of patterns. The comparison consists of calculating the difference in the percentage of black pixels between the digit being examined and the specific pattern. The difference is calculated for all six fields, i.e., A11–A32. Then, the absolute value of the difference obtained for a specific field is subtracted from the number 1000. The results obtained from all fields are added together and, as a result, a checksum for a given pattern is obtained. In the case of perfect compliance with the standard, the checksum is 6000. An example of the comparison of the A11 fields for the number 7 with the number 1 digit looks as follows:

(1) $$A\prime_{11}\left(7\right)=A_{11}\left(7\right)-A_{11}\left(1\right)$$

(2) $$A\prime_{11}\left(7\right)=274-0=274$$

(3) $$S_{11}=1000-A\prime_{11}\left(7\right)$$

(4) $$S_{11}=1000-274=726$$

(5) $$A\prime_{12}\left(7\right)=A_{12}\left(7\right)-A_{12}\left(1\right)$$

(6) $$A\prime_{11}\left(7\right)=652-0=652$$

(7) $$S_{12}=1000-A\prime_{12}\left(7\right)$$

(8) $$S_{12}=1000-652=348$$

where $S_{12}$ is the partial checksum for field *A*_12_, and *A*’_12_ is difference in the percentage between the displayed 7 and the number 1 digit multiplied by 1000.

Similarly, partial checksums are calculated for all fields, and the final checksum value for comparison of the display digit (7) with the number 1 digit is the sum of all six subtotals. In this example, the checksum calculated from the comparison of the number 7 with the pattern of the number 1 is 5230. The checksum for comparing the number 7 from the display with the pattern in the example under consideration was 5960. The recognized character is compared to all patterns and the result is the one that obtains the largest checksum value.

While the program is running, it may turn out that the currently recognized image is in a transient state, most often as a result of the display element lighting up. Due to the image recognition algorithm used in this situation, the program compares the obtained image with the standards and always retrieves the result with the largest checksum, even though there is no specific digit on the display. For example, the lack of indication is most similar to the pattern of the number 1. In order to eliminate the error associated with the transient state of the display or the incorrectly displayed elements, additional protection is introduced. The criterion for eliminating accidental erroneous readings is defined as the minimum value of the control sum that the recognized element must obtain for the result of this recognition to be considered reliable. As a result of the tests carried out on the various types of measuring instruments, this criterion was set at 5300. Due to the very wide variety of instruments, this criterion was entered in the program only as the default value. If the person performing the initial configuration of the program for a specific type of device finds that this value should be different, he can change it and save it in the configuration file for this type of device. If, during the calculation process, the criterion is not met for at least one of the characters, the entire result is considered unreliable and rejected. Due to the short recognition time of an individual millisecond, the described method does not affect the usability of the program. By default, the recognition results are saved to a file every 300 ms. During this time, at least a dozen or so complete program loops are carried out and the appearance of individual results that do not meet the criterion does not change the final results saved to the file. By using this criterion in this research, it was possible to achieve 100% recognition efficiency at 300 ms to save the results.

A comparison with the other methods published in the literature shows that the method developed for the automated calibration of multimeters is fast enough and ensures 100% repeatability of the results obtained.

Recognition and interpretation of the image allows to eliminate human participation from the tested instrument during reading. In order to eliminate human participation, and thus to accelerate the calibration process, there are many commercial programs required to set further measured values of the physical quantity from the calibrator, such as MET/CAL and Calpro 101. They are ready and closed programs prepared for use in accordance with the requirements of their manufacturer.

To implement the developed image recognition method in an easy, fast, and effective manner in metrological practice, the most popular software in laboratories, which is Microsoft Excel with built-in Visual Basic for Applications (VBA) language, was used. The use of the MS Office environment due to its easy availability allows for wide practical application of the developed method. Implementation of the solution in subsequent sheets created for new types of instruments does not require the performer of this task to have either knowledge of the OCR Digit Reader or the basics of programming in VBA.

**Figure 6:**
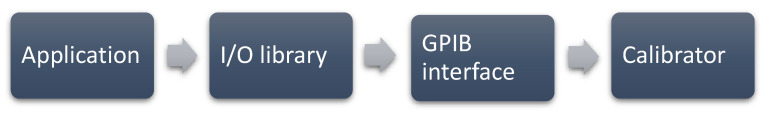
Block diagram of calibrator control using Virtual Instrument Software Architecture (VISA) input/output (I/O) libraries.

The calibrator is controlled using input/output (I/O) libraries and the Virtual Instrument Software Architecture (VISA) standard. A collection of Agilent I/O Libraries Suite libraries and utilities is available on the producer’s website: www.keysight.com. The I/O library is a set of program procedures responsible for carrying out typical operations within a specific interface platform. The purpose of using this program layer is to free the application designer from the details of operating a specific interface platform by providing ready-made, universal functions that allow the application to communicate with the selected measuring device. I/O libraries (i.e., SICL, VISA, and VISA COM) enable the device to communicate in various programming environments (Agilent VEE Pro, Microsoft Visual Studio, etc.) that are compatible with GPIB, USB, LAN, RS-232, PXI, AXIe [35]. VISA is a standard that allows communication between a PC and a measuring device. This standard allows sending commands and reading results from laboratory and measuring devices, such as power supplies, calibrators, oscilloscopes, multimeters, and many others. Most modern instruments support USB, LAN, GPIB, and PCI/PCIe connections, and at the lowest level, each of these interfaces handles data and communication in a different way. This situation is greatly simplified when using the middle tier that deals with managing I/O interfaces. As a result, there is no need to place commands for various types of connections in the application itself, which greatly facilitates the writing of software and standardizes communication with measuring instruments [36]. The scheme of operation of the software based on I/O libraries is shown in Figure 6.

**Figure 7:**
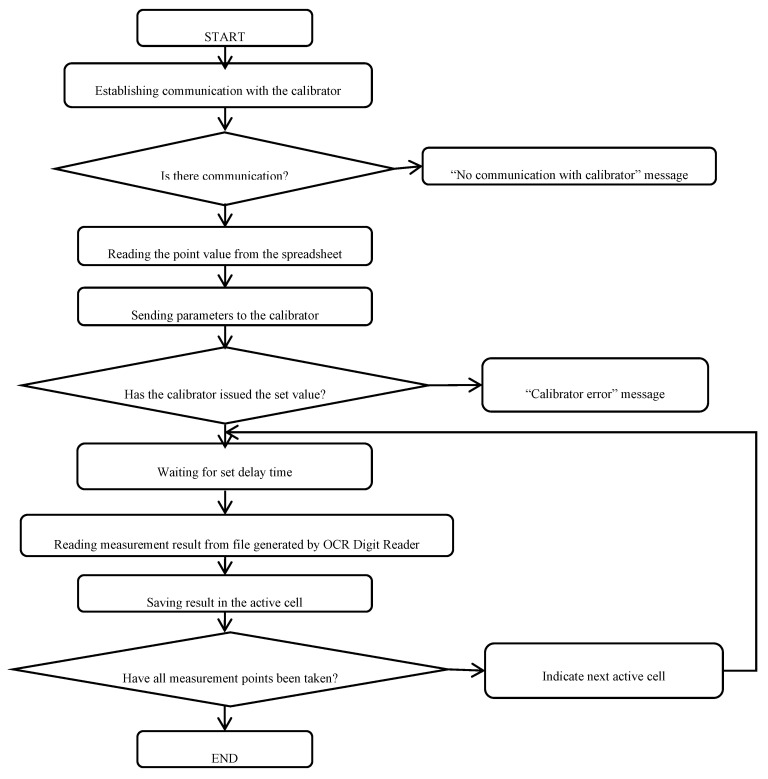
The algorithm of the calibrator control software.

The sheet works by sending a command to the calibrator with the appropriate value of a given physical quantity called. The calibrator issues the set value, and after the set time elapses, the data from the file with the measurement results are read and the value is saved in the appropriate cell. The delay time is selected individually for individual measurement quantities, and differences in delay times result from the characteristics of the multimeter being tested. The appropriate determination of these characteristics allows to significantly reduce the time of the metrological service, while ensuring the stability of the measurements. Selection of the delay time before taking the first reading is necessary to eliminate errors that occur when the instrument is in a transient state. The differences in the times of reaching the steady state differ significantly, both for individual physical quantities and due to the design parameters of a given type of device. For example, the time to settle the result for a FLUKE 117 multimeter when measuring DC voltages is less than 1 s when the time needed to stabilize the result when measuring resistance over the MΩ range is already about 10 s. Delay times are selected empirically and recorded in a standard spreadsheet for a specific type of measuring instrument. The whole process is repeated for subsequent measuring points. The operating principle of calibrator control and saving measurement results are schematically presented in the form of an algorithm in Figure 7.

The proposed solution is partly automated. The operator’s only tasks are to physically connect the calibrator to the multimeter using test leads, to change the ranges and measured physical quantities on the tested multimeter, and to start the procedure with the “START” button. Displaying the values on the calibrator and entering the read results into the appropriate cells of the table is done automatically.

## 3. Results

**Figure 8:**
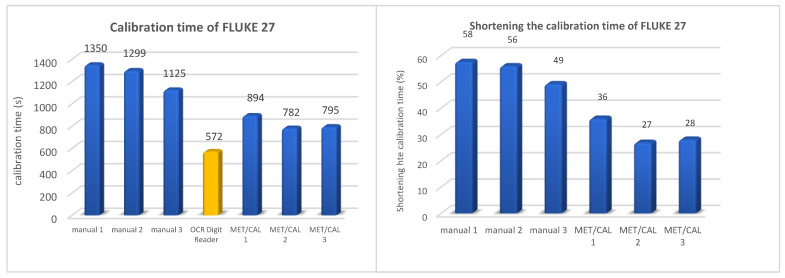
Comparison of the calibration methods for FLUKE 27 multimeter.

To compare the proposed solution to the method currently used, a full calibration of several types of multimeters was performed, selecting the measurement points in accordance with the applicable methodologies. The detailed results obtained with the help of an automated measuring station are presented using the calibration of a FLUKE 27 multimeter as an example. The basis for determining the measurement points was the PP-07.10.01-2-2018-1WOM-multimetry_cyfrowe procedure, which was written based on References [37,38]. Calibration of multimeters was performed manually by three different people using a spreadsheet to place the results, perform the calculations, and create a final report. The second method used for comparison was also the widely used semi-automatic method based on the MET/CAL software. In this method, the technician performing the calibration does not need to specify specific output quantities from the calibrator himself—this is done by the control software. However, writing the results from the display of the calibrated multimeter is done by a technician. In this case, three calibrations were also carried out by three different people. The last method was the automatic calibration method described in this article using the OCR Digit Reader software. The time for performing full calibration with all three methods is shown in Figure 8.

Based on the tests, it was confirmed that the full calibration procedure for the FLUKE 27 multimeter was reduced at an average of 54% compared to manual calibration. Compared to the semi-automatic method based on the MET/CAL software, the average time reduction was 33%.

**Figure 9:**
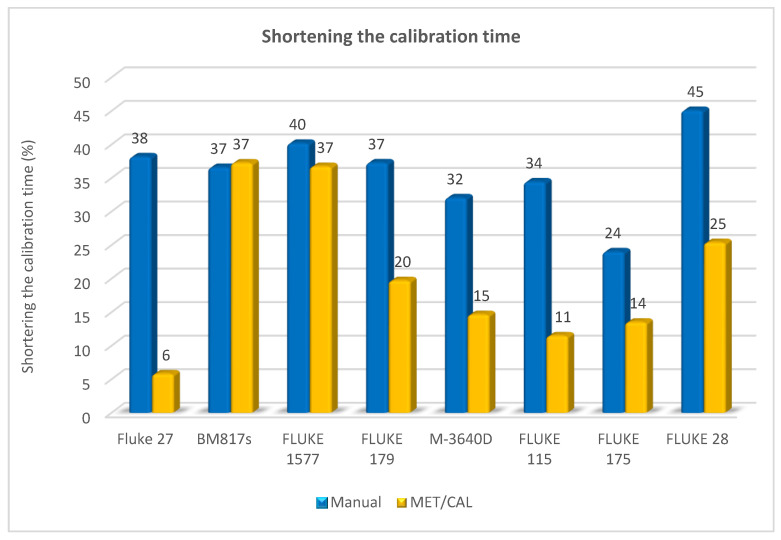
Comparison of the shortened calibration time for different types of multimeters.

In order to confirm the effectiveness of the proposed solution, other types of multimeters were tested in an analogous way. The results of these tests are presented in the form of a graph in Figure 9.

The tests carried out on different types of multimeters confirmed the effectiveness of the proposed method. In each of the tested multimeters, the calibration time was shortened, both in comparison with the manual and semi-automatic methods based on the MET/CAL software. 

## 4. Discussion

Based on this research, it was possible to confirm the effectiveness of the proposed method. The use of dedicated software to recognize the result from a seven-segment display allowed to automate the calibration process of a large group of devices that are not equipped with a communication interface. The proposed solution is simple in its practical implementation. It does not require people to prepare reports of specialized knowledge for subsequent types of measuring instruments. The tests confirmed its 100% effectiveness in recognizing the result with the help of the OCR Digit Reader software.

What is new?

The proposed solution is novel and thus far not used in laboratory practice. The authors did not come across a solution that would allow automation of the calibration process of multimeters not equipped with a communication interface, neither in the literature nor in professional practice. Although image recognition software is widely used, the use of dedicated image recognition software from a seven-segment display in conjunction with calibrator control software is an innovative solution suitable for wide application in everyday laboratory practice. The obtained results confirm the effectiveness of this solution. However, it should be remembered that the resulting time reduction refers to the calibration of subsequent pieces of a given type of multimeter.

At this stage, the software has several limitations. The maximum number of recognized digits is eight. This is due to the design solutions of the multimeters currently available on the market. Another limitation is the need to manually configure the reading fields and filter settings. These are restrictions that do not prevent one from fully implementing the proposed solution in practice. In order to facilitate work with this software, research was carried out to automatically recognize the reading fields and to choose their size. Now, the first calibration of a new type requires program configuration, which takes 5–10 min.

At present, during typical calibration, the uncertainty estimation principles described in Reference [31] are applied. The uncertainty budget for the calibration of the digital multimeter when making one measurement at each point is as follows:

Only type A uncertainty is taken into account. The error of indication *E_X_* of the digital multimeter (DMM) to be calibrated is obtained from:

(9) $$E_{x}=V_{ix}-V_{S}+\delta V_{ix}-\delta V_{S}$$

where *V_iX_* is the voltage, indicated by the DMM (index *i* means indication); *V_S_* is the voltage generated by the calibrator; *δV_iX_* is the correction of the indicated voltage due to the finite resolution of the DMM; and *δV_S_* is the correction of the calibrator voltage due to drift since its last calibration, to deviations resulting from the combined effect of offset, non-linearity, and differences in gain, to deviations in the ambient temperature, and to deviations in the mains power loading effects resulting from the finite input resistance of the DMM to be calibrated.

Expanded uncertainty:

(10) $$U=k\cdot u\left(E_{X}\right)$$

where *U* is the expanded uncertainty, *u* is the standard uncertainty, and *k* is the coverage factor.

**Figure 10:**
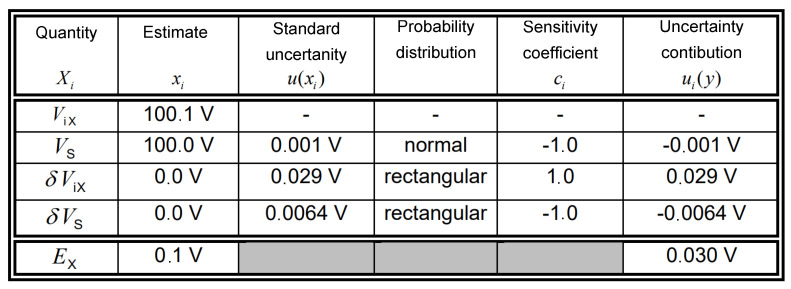
Uncertainty budget (*E_x_*).

In typical laboratory practice, the extension coefficient is rigidly assumed as the value of *k* = 2, which corresponds to the normal distribution, or *k* = 1.65, which corresponds to the rectangular distribution of standard uncertainty. Figure 10 presents the uncertainty budget for the calibration of the digital multimeter given as an example in Reference [37].

As can be seen, the components *δV_iX_* and *δV_S_* have a rectangular distribution and the *V_S_* component has a normal distribution. This is typical during calibration, and assuming a rigid value for the expansion factor leads to smaller or larger inaccuracies when estimating uncertainty. The proposed solution also includes the option of calculating the extension factor as follows.

The measurement results after calibration are given in accordance with the recommendations for calibration laboratories, with expanded uncertainty for a confidence level of *p* of approximately 95%. For such an assumption, a mathematical condition can be formulated in the form:

(11) $$\int_{-U}^{U}g\left(y\right)\cdot dy=p≅95\%$$

where:

(12) $$g\left(y\right)=g_{1}\left(x_{1}\right)*\ldots *g_{N}\left(x_{N}\right)$$

where *g(y)* is the probability density function of the measured value.

The above equation can only be solved numerically by performing a multiple-convolution mathematical operation of the input probability density function. In the case when the input quantities are described only by means of normal and rectangular distributions, its convergence with the PN-type distribution can be used to describe their weave. A PN distribution can be called a distribution that is a weave of a single rectangular and normal distribution. Then the equality is met:

(13) $$g\left(y\right)=g_{PN}\left(x\right)$$

where *g_PN_(y)* is the probability density function of PN.

The PN distribution is characterized by a probability density function:

(14) $$g_{PN}\left(x\right)=\frac{1}{2\sqrt{6\pi }\cdot r}\int_{x-\sqrt{3}\cdot r}^{x+\sqrt{3}\cdot r}exp\left[-\frac{z^{2}}{2}\right]dz$$

where *r* is a distribution parameter that defines the ratio of standard deviations of the rectangular distribution and the normal distributions forming it, defined as the quotient:

(15) $$r=\frac{\sigma_{P}}{\sigma_{N}}$$

where $\sigma_{P}$ is the standard deviation of the rectangular distribution and $\sigma_{N}$ is the standard deviation of the normal distribution. The expansion coefficient for the distribution is determined by the function:

(16) $$k_{PN}=f\left(r,p\right).$$

**Figure 11:**
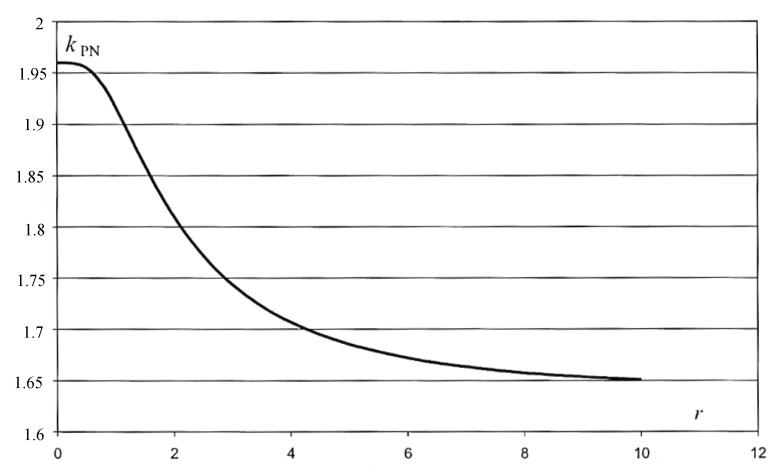
Probability density function of the PN-type distribution [39].

The $k_{PN}$ function does not have an analytical form, but can be determined numerically. For the confidence level *p* = 95%, the coverage factor assumes values between 1.6443 and 1.96. The value of 1.96 is the value taken by the distribution in the case of a definite dominance of the inbound component from the normal distribution. We are then dealing with a situation typical for calibration, where the value of factor 2 is assumed for a range of about 95% and the normal probability distribution of the initial quantity. A graph illustrating the change in the value of the expansion coefficient for the PN distribution depending on the value of the distribution parameter *r* is shown in Figure 11.

Assuming that the distribution of the measured quantity is approximated by means of the PN distribution, the *r* parameter of this distribution should be determined. Knowledge of this parameter will allow the determination of the extension coefficient value for a given confidence level. The distribution parameter is determined using an approximation:

(17) $$r=\frac{\left|u_{i}\left(y\right)\right|}{\sqrt{u_{c}^{2}\left(y\right)-u_{i}^{2}\left(y\right)}}$$

where $u_{i}\left(y\right)$ is the largest share in the uncertainty of the composite input quantity with a rectangular distribution, and $u_{c}\left(y\right)$ is the total value of the standard uncertainty.

In optimization calculations, the factor resulting from the finite resolution of the tested device is used as the largest share of uncertainty with a rectangular distribution. The method of determining the *r* parameter uses the principle that the distribution of the output quantity converges to the PN distribution regardless of the number of input quantities to which the rectangular and normal distributions have been assigned. On the other hand, the *r* parameter of the PN distribution is determined by the measured quantity component of the rectangular distribution and the largest share among all rectangular components. Based on the system of components in the uncertainty budget, the *r* parameter should be determined, followed by the extension factor. The method consists of the approximation of the unknown coefficient of expansion with the coefficient for distribution: normal, trapezoidal, and rectangular [40]. The selection of the appropriate distribution depends on the value of the $r_{u}$ parameter. This can be written as:

(18) $$k=k_{N}\;dla\;0<r<1$$

(19) $$k=k_{T}\;dla\;1\leq r\leq 10$$

(20) $$k=k_{P}\;dla\;r>10$$

where $k_{N}$ is the extension factor for normal distribution; $k_{T}$ is the extension factor for trapezoidal distribution; and $k_{P}$ is the extension factor for rectangular distribution.

For the calculation of the expanded uncertainty during calibration, the extension factor for the individual distributions with an assumed confidence interval of 95% assumes the values:

(21) $$k_{N}≅2$$

(22) $$k_{T}=\sqrt{\frac{3}{r^{2}+1}\left(1+r-2\sqrt{r\left(1-p\right)}\right)}$$

(23) $$k_{P}=\sqrt{3}p≅1.65.$$

Using this solution in a spreadsheet, the uncertainty estimation function takes the form of:

(24) $$U\left(y\right)=\sqrt{\frac{3}{r_{u}^{2}+1}\left(1+r_{u}-2\sqrt{r_{u}\left(1-p\right)}\right)}\sqrt{\overset{N}{\underset{i=1}{\sum }}{\left(\frac{\partial f}{\partial x_{i}}\right)}^{2}u_{i}{}^{2}\left(x_{i}\right)}.$$

Thanks to this method, uncertainty estimation is definitely more precise, which can and often affects the metrological assessment of the device.

The stand presented herein allows to shorten the calibration time. The next stage is to introduce a series of measurements at each measuring point, which becomes possible due to a significant reduction in the measurement time. Work is underway to apply multi-criteria optimization methods to determine the optimal number of measurements in a series. The introduction of a series of measurements will significantly improve the quality of the results obtained by reducing the measurement uncertainty and by increasing the precision of the results obtained as the average of the measurement series.
